# Residual monomers and volatile leachable substances of conventional and CAD/CAM-fabricated denture base materials — GC-MS analysis and surface characterization

**DOI:** 10.1007/s00784-026-07224-9

**Published:** 2026-10-21

**Authors:** Viktor Martian, Christoph Sasse, Ralf Bürgers, Andrea Schubert, Oskar Bunz

**Affiliations:** Department of Prosthodontics, University Medical Center Göttingen, Göttingen, 37075 Germany

**Keywords:** Denture base materials, Methacrylic acid esters, Residual monomers, CAD/CAM, Gas chromatography–mass spectrometry

## Abstract

**Objectives:**

Methacrylic acid ester-based resins for denture fabrication release residual monomers and leachable substances that may interact with oral tissues. With increasing adoption of CAD/CAM technologies, a systematic comparison of leachable substance profiles and surface characteristics across polymerization methods is needed.

**Materials and methods:**

This study compared the chemical composition and profiles of leachable substances of four denture materials – autopolymerized, thermal polymerized, milled, and 3D-printed – using gas chromatography–mass spectrometry (GC–MS). Surface roughness was evaluated by confocal microscopy.

**Results:**

GC–MS screening detected 24 distinct compounds in the solid specimens. Among the MMA/PMMA-based materials, methyl methacrylate content was highest in the thermally polymerized resin, followed by the autopolymerized and milled materials, and absent from the 3D-printed resin, consistent with its MMA-free formulation. MMA levels in the extracts did not exceed those of the negative control in any group. Surface roughness was comparable on the polished face (R_a_ ≈ 0.21–0.25 μm), consistent with SEM findings but material-dependent on the unpolished, tissue-contacting face (R_a_ 0.37–1.03 μm).

**Conclusions:**

Both fabrication method and material formulation influence the volatile and semi-volatile extractables of denture resins. As the present GC–MS screening may not reliably capture high-molecular-weight dimethacrylates such as Bis-EMA and UDMA, novel CAD/CAM materials require additional, complementary toxicological evaluation.

**Clinical relevance:**

The leachable substance profiles of CAD/CAM denture resins, particularly the distinct compounds detected in the 3D-printed material, underscore the need for material-specific biocompatibility data to support informed clinical material selection and patient safety.

**Supplementary Information:**

The online version contains supplementary material available at https://doi.org/10.1007/s00784-026-07224-9.

## Introduction

Polymethyl methacrylate (PMMA) and related methacrylic acid ester-based materials have been cornerstone materials in prosthodontics since their introduction in 1937 [1], primarily for the fabrication of complete and partial removable dentures and occlusal splints [2]. PMMA is synthesized through the polymerization of methyl methacrylate (MMA), typically initiated by heat, light, or chemical activators [3–5]. Traditional methods of methacrylic acid ester processing include thermal polymerization and autopolymerization [6], whereas recent advancements have introduced computer-aided design/computer-aided manufacturing (CAD/CAM) techniques, including milling of prepolymerized blanks and 3D printing of photopolymerizable resins [7]. Beyond conventional PMMA, modern denture polymers increasingly incorporate alternative methacrylate monomers, such as bisphenol A glycidyl dimethacrylate (Bis-GMA), ethoxylated bisphenol A dimethacrylate (Bis-EMA), and urethane dimethacrylate (UDMA), offering improved mechanical and handling characteristics, particularly in light-cured composite restorative resins [8, 9].

Despite their widespread use, concerns regarding the biocompatibility of methacrylic acid ester-based materials persist due to the incomplete monomer conversion during polymerization [10, 11]. Residual monomers and other leachable substances such as formaldehyde [12, 13], phthalates [14] and for soft linings, plasticizers [15, 16] can diffuse into saliva and interact with cells of the oral mucosa in vivo [11], potentially triggering local inflammatory responses and systemic effects [17]. Incomplete conversion of polymers is primarily attributed to steric hindrance within the growing polymer network, which traps unreacted monomers [18, 19]. Notably, the residual monomer content varies widely depending on the polymerization method, with heat-polymerized resins generally showing lower leachability compared to self-curing counterparts [20–22]. CAD/CAM milled dentures are presumed to exhibit greater mechanical and chemical stability due to industrial prepolymerization under controlled conditions [23, 24]. Conversely, 3D-printed resins offer high precision and customization [25]. However, depending on the printing system and post-cure protocol, they may exhibit lower degrees of conversion and consequently higher leachability [26].

Biocompatibility is critical for all dental biomaterials, particularly for those in continuous contact with the oral mucosa [2]. Residual monomer exposure has been associated with clinical symptoms including mucosal irritation, ulceration [27], and erythema [28]. With the rising adoption of CAD/CAM technologies in prosthodontics, there is a growing need to reassess the biocompatibility of novel denture base materials produced by milling and 3D printing, as these materials may differ substantially from conventional formulations in composition, degree of polymerization, and additive content, potentially resulting in material- and fabrication-specific profiles of leachable substances [29, 30]. A better understanding of how polymerization method and material formulation affect residual monomer content and leachability is therefore essential for optimizing both the safety and clinical performance of denture materials.

The aim of this study was to evaluate the chemical composition and leachable substance profile of two CAD/CAM-fabricated (milled and 3D-printed) and two conventionally processed methacrylic acid ester-based denture materials. We hypothesized that polymerization method and material formulation shape the chemical composition of the cured material and the spectrum of leachable substances, with CAD/CAM-fabricated materials releasing fewer methacrylate monomers than conventionally processed resins.

## Materials and methods

### Specimen preparation

Two CAD/CAM-fabricated and two conventionally processed denture base resins were included in this study. For the CAD/CAM groups, cylindrical specimens (⌀ 10 mm) were produced from two different resin systems. Specimens of the prepolymerized resin (**PreP**; Temp Basic Tissue, Zirkonzahn, Italy) were milled from prefabricated resin blanks using a computer numerical control (CNC) milling unit (M5; Zirkonzahn, Italy). Specimens of the photopolymerized resin (**PP**; Denture Base Resin OP, Formlabs, Somerville, MA, USA) were fabricated by stereolithography (Form 3B+; Formlabs, Somerville, MA, USA). After printing, specimens were washed in isopropanol for 15 min (Form Wash; Formlabs, MA, USA) and dried with compressed air. Post-polymerization was carried out by placing specimens in a Petri dish containing glycerol heated to 80 °C and exposing them to near-ultraviolet light (405 nm) for 60 min (Form Cure; Formlabs, MA, USA), in accordance with the manufacturer’s instructions for use. For the conventionally processed groups, cylindrical specimens (⌀ 10 mm) of the autopolymerized resin (**AP**; PalaXpress, Kulzer, Germany) and the thermally polymerized resin (**TP**; Paladon 65, Kulzer, Germany) were self-produced using the remaining rings of the prepolymerized (PreP) blanks. The rings were closed with thermoforming polymer sheets and dental wax, embedded in dental plaster within a dental cuvette, and the wax was subsequently removed to form an injection channel. Following the manufacturer’s instructions, the resin powder and liquid monomer were combined and injected into the blank rings via an injection unit (Palajet^®^, Kulzer, Germany) at 5 bar pressure. After a 15 min waiting period, polymerization was carried out in a hot water bath unit (Palamat^®^ elite, Kulzer, Germany) at 2 bar: the autopolymerized resin was cured at 55 °C for 30 min, and the thermally polymerized resin at 70–100 °C for 2 h (long curing cycle). After cooling, the plaster was removed and specimens were brought to final dimensions by CNC milling.

The rods of all four material groups were cut into cylindrical specimens (⌀ 10 mm, height 2.5 mm) using an automated cutting machine (Micracut 201; Metkon Instruments, Bursa, Turkey) at 1500 rpm and a feed rate of 50 μm/s, under continuous water irrigation/cooling to prevent thermal damage and heat-induced monomer loss. Specimen dimensions were selected in accordance with DIN EN ISO 10993-12 [31].

One side of each specimen was polished with silicon carbide abrasive paper (grit sizes P800, P1200, and P2000) following an established protocol [32] at 50 rpm and a 5 N applied force. The opposing surface was intentionally left as-cut to mimic the tissue-contacting palatal aspect of a denture. As specified by the manufacturer, conventional materials required post-fabrication immersion in lukewarm water to reduce residual monomer content. Accordingly, AP and TP specimens were submerged in a large glass Petri dish filled with water at 30 °C for 4 h per side. All specimens were stored in a dry, dark environment.

### Surface characterization

Surface roughness (arithmetic roughness average, $\:{R}_{a}$) was evaluated following a previously described protocol [32]. Briefly, five specimens per material group were cleaned in isopropanol; R_a_ was then measured on both the polished and the unpolished (tissue-side) surface at three locations per specimen per surface (*n* = 5 specimens per material) using 20× magnification by confocal microscopy (Smartproof 5; Zeiss, Oberkochen, Germany) and automated analysis (ConfoMap ST, Zeiss, Oberkochen, Germany).

### Extract preparation

Extracts from PreP, PP, AP, and TP specimens (*n* = 14 specimens pooled per group in a single extraction batch) were prepared in compliance with DIN EN ISO 10993-5/12 [31, 33] and established literature [13, 34, 35], applying a surface-area-to-volume ratio of 3 cm²/ml. The total cylindrical surface area was calculated as 2 × *π* × r × (r + h) = 2 × *π* × 5 mm × (5 mm + 2.5 mm) = 2.356 cm², yielding an extraction volume of 0.785 ml per specimen. Specimens were UV-C-sterilized (254 nm) on autoclaved glass dishes for 20 min per side (HERAsafe™ KS 12; Life Technologies GmbH, Darmstadt, Germany). Subsequently, 14 specimens were incubated in a closed autoclaved glass bottle containing 11 ml of Keratinocyte Growth Medium 2 with supplements (KGM+; PromoCell, Heidelberg, Germany) on a rocker for 48 h at 37 °C to simulate clinically relevant extraction conditions. Pure KGM+ served as a negative control in all subsequently performed experiments. After sterile filtration (0.2 μm), extracts were aliquoted into autoclaved amber glass vials and stored at −20 °C.

### SEM images

Surface morphology was examined by scanning electron microscopy (SEM; JEOL JSM-IT500, JEOL Germany GmbH, Freising, Germany). Prior to imaging, specimens were sputter-coated with a gold-palladium alloy to ensure adequate electrical conductivity. Images were acquired at an accelerating voltage of 10 kV and a magnification of ×500 using secondary electron detection (SED). Focus and image sharpness were optimized for each specimen prior to image acquisition.

### Gas chromatography–mass spectrometry (GC–MS)

A semi-quantitative gas chromatography–mass spectrometry (GC–MS) screening of the denture resins, their extracts, and the extraction medium (negative control; pure KGM+) was conducted (DEKRA Laboratory for Material Testing and Damage Analysis, Bretten, Germany). For material extraction, 2 g of each material (12 specimens per group) were extracted by ultrasonication in dichloromethane (DCM) for 2 h at 50 °C. The DCM-soluble polymer part was precipitated with n-hexane and filtered through a syringe filter (0.2 μm/PTFE). Fifteen milliliters of each extract and the extraction medium were transferred to separate separatory funnels and liquid–liquid extracted four times with 20 ml of DCM at 23 °C. The resulting organic DCM phase was dried over anhydrous sodium sulfate, concentrated to a volume of 2 ml, and transferred quantitatively to a 5 ml volumetric flask. An aliquot of each final extract was mixed with an internal standard (toluene-D8) and injected into the GC–MS system for analysis. The autosampler AOC-20i Plus (Shimadzu, Duisburg, Germany) was operated with an injection volume of 1 µl, a split ratio of 10:1, and an injection temperature of 300 °C. The GC was equipped with a HP Ultra 2 column (L = 50 m, ID = 0.32 mm, df = 0.52 μm); helium was used as the carrier phase at a linear velocity of 40 cm/s. The mass spectrometer GC–MS QP2020 NX (Shimadzu Duisburg, Germany) was operated with an interface and ion source temperature of 280 °C and a scan range of 32–500 m/z. Compounds were annotated from the resulting total ion chromatograms (TICs) by comparison with the National Institute of Standards and Technology (NIST) 2019 mass-spectral library and quantified as toluene-D8 equivalents in a semi-quantitative manner [36]. All compound identities reported here are tentative annotations based on library matching of electron ionization mass spectra. Signals were additionally reported as unassigned when they could not be attributed to a declared constituent, a known impurity, or a known degradation product of the respective material; for these, the library name is retained for traceability only and is not asserted as an identification. The visualization of the TICs and the individual peaks of MMA was performed using OpenChrom software version 1.6.13.

### Use of artificial intelligence tools

During the preparation of this manuscript, the authors used a large language model (Claude Sonnet 5; Anthropic, San Francisco, CA, USA) to assist with language editing, grammatical correction, and stylistic revision of the manuscript text. The AI tool was not used for data analysis, interpretation of results, or generation of scientific content. All AI-assisted revisions were reviewed and approved by the authors, who take full responsibility for the accuracy and integrity of the published work.

## Results

### Surface characterization

All polished specimens exhibited comparable surface roughness, with mean $\:{R}_{a}$ values ranging from 0.21 μm (SD: 0.02) for AP to 0.25 μm (SD: 0.03) for PP. On the unpolished face (*n* = 5 per group), mean $\:{R}_{a}$ values were 1.03 μm (SD: 0.45) for PreP, 0.98 μm (SD: 0.39) for AP, 0.47 μm (SD: 0.29) for TP, and 0.37 μm (SD: 0.09) for PP, with the unpolished PreP surface approximating the rough palatal aspect of clinical dentures and thereby representing a clinically realistic condition.

### SEM images

**Fig. 1:**
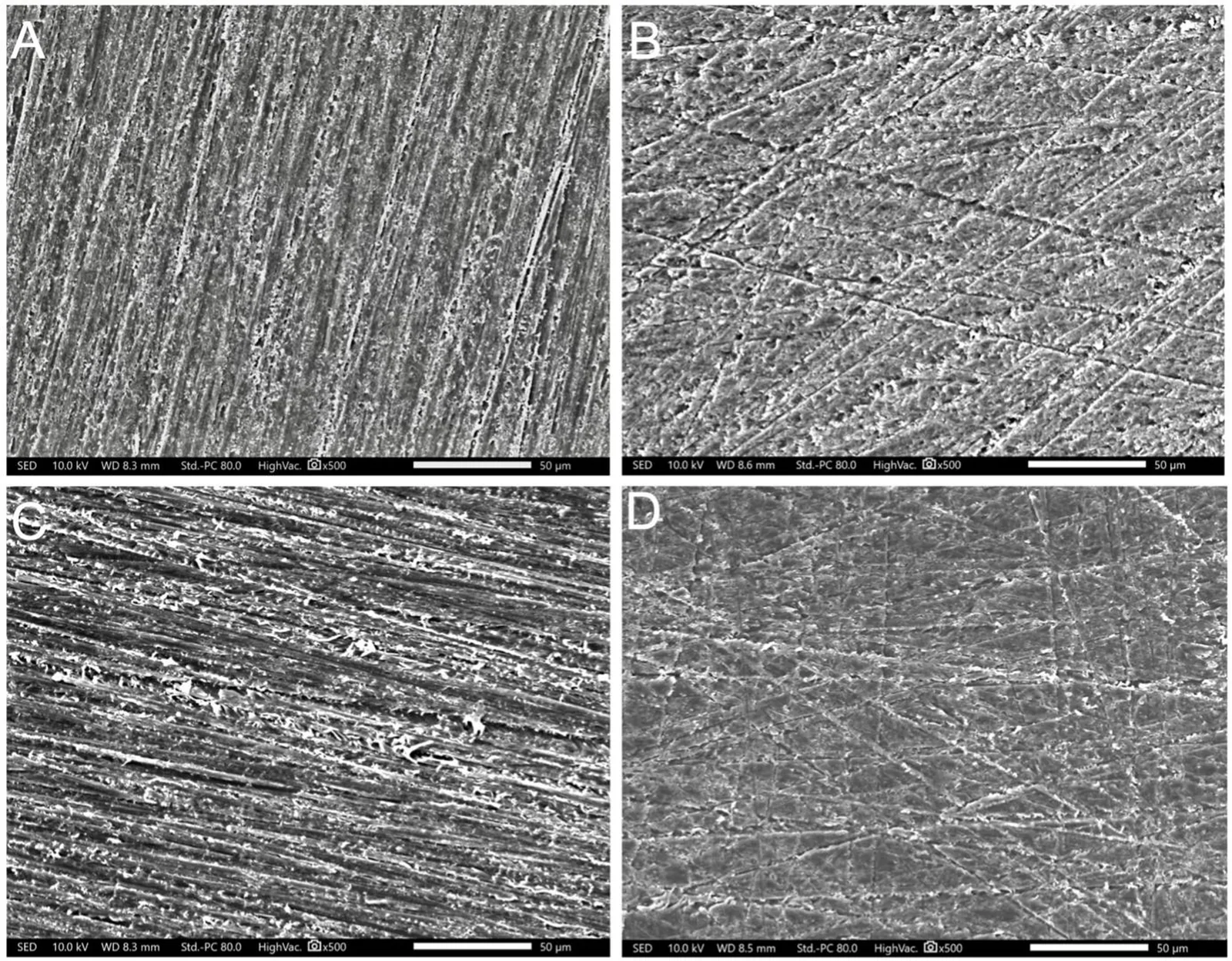
Exemplary scanning electron microscopy (SEM) images of the four denture base resin specimens at ×500 magnification (scale bar = 50 μm). (**A**) Prepolymerized resin (PreP). (**B**) Photopolymerized resin (PP). (**C**) Autopolymerized resin (AP). (**D**) Thermally polymerized resin (TP). Detailed morphological descriptions are provided in the Results

SEM analysis revealed distinct surface morphologies among the four polished resin surfaces. The prepolymerized CAD/CAM resin (PreP) exhibited a predominantly unidirectional surface topography (Fig. 1A). The photopolymerized resin (PP) displayed a markedly different surface texture, featuring an irregular, multidirectional pattern of fine intersecting grooves arranged in an approximately orthogonal network (Fig. 1B). The autopolymerized resin (AP) showed a surface dominated by pronounced, coarse, and densely packed unidirectional grooves with considerable amounts of loosely adherent particulate debris accumulated within and along the groove paths (Fig. 1C), suggesting a less cohesive surface structure than PreP. The thermally polymerized resin (TP) presented a complex multidirectional groove pattern with crossing scratch-like structures of varying depth and orientation, resulting in an overall moderately rough and heterogeneous topography (Fig. 1D).

### GC–MS analysis

**Fig. 2:**
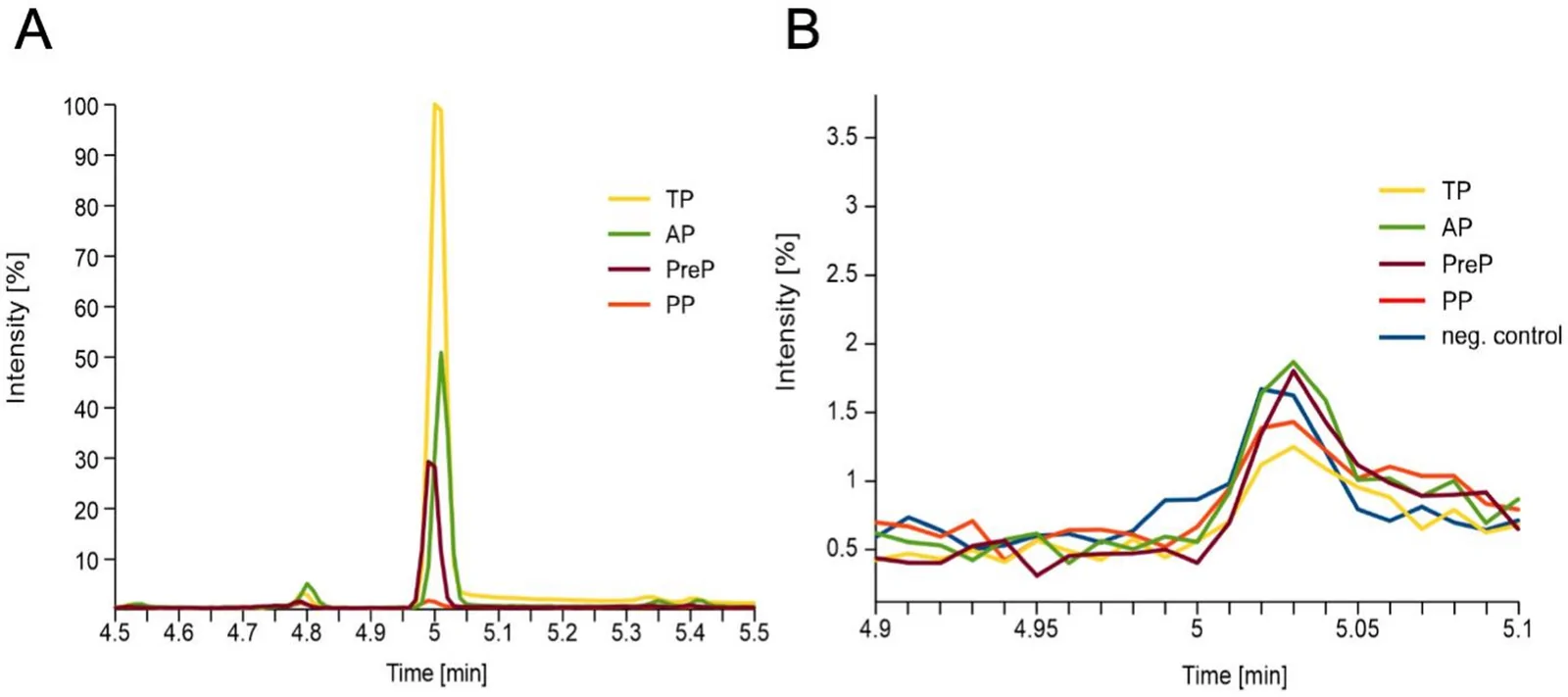
Analysis of the MMA amount in solid materials and the corresponding extracts. Relative intensity of the MMA amount received from solid samples (**A**) and from the extracts (**B**). The measurements show that the highest relative MMA signal was detected in the TP sample and the lowest in the PreP sample. No MMA was detected in the PP material. In contrast, no elevated levels of MMA were detected in any extracts compared to the negative control consisting of pure KGM+ medium

A semi-quantitative gas chromatography–mass spectrometry (GC–MS) screening of the PreP, PP, AP, and TP solid specimens and their corresponding extracts was performed. Analysis of the solid specimens revealed a total of 24 distinct compounds in the total ion chromatograms (TICs) (Figure S1, Table 1). Among the detected methacrylate esters, methyl methacrylate (MMA) was detected in PreP (2,254 mg/kg), AP (4,040 mg/kg), and TP (11,068 mg/kg) specimens, but was not detected in PP specimens, in line with its MMA-free, dimethacrylate-based formulation (Fig. 2A). All values are semi-quantitative mass fractions expressed as toluene-D8 equivalents. Thermal polymerization (TP) yielded the highest MMA content in the solid material, followed by autopolymerized (AP) and prepolymerized (PreP) specimens, while photopolymerized (PP) specimens contained no detectable methyl methacrylate, consistent with its manufacturer-declared Bis-EMA-, UDMA-, and TMPTMA-based formulation. Although MMA was detected in the solid material of the TP, PreP, and AP samples, GC-MS analysis of the corresponding extracts showed that the MMA concentrations were at or below the level of the negative control (pure KGM+) (Fig. 2B).

In addition, methyl isobutyrate was detected at similarly low levels in AP (42 mg/kg) and TP (46 mg/kg) specimens. Of note, isobutyl methacrylate was detected at considerably higher concentrations: 412 mg/kg in AP and 7,840 mg/kg in TP specimens.

Beyond the methacrylate esters, the largest single finding was tributyl acetylcitrate in AP (5,185 mg/kg), a plasticizer that exceeded the MMA content of this material and is not among the manufacturer-declared ingredients, followed by phenyl benzoate in TP (799 mg/kg); all further compounds were detected at mass fractions between 30 and 653 mg/kg (Figure S1, Table S1).

Three low-abundance signals, a C_6_H_12_O species in AP (81 mg/kg), a C_4_H_6_O_3_ species in TP (98 mg/kg) and an N-/S-containing species in AP (30 mg/kg), could not be assigned with confidence and are reported as unassigned (Table 1); the corresponding entries in Figure S1 retain the library annotations as delivered by the analytical laboratory.

**Table 1:** GC–MS-detected compounds of the four tested denture base materials

Category	Compound	Formula	PreP	PP	AP	TP
Poly-/monomer	Methyl methacrylate (MMA)	C_5_H_8_O_2_	+	−	+	+
	2-Propenoic acid, 2-methyl-, 2-methylpropyl ester (isobutyl methacrylate)	C_8_H_14_O_2_	−	−	+	+
	Hexanedioic acid, 2-methyl-5-methylene-, dimethyl ester	C_10_H_16_O_4_	+	−	−	−
Initiator/Degradation product	Benzoic acid, 2,4,6-trimethyl-	C_10_H_12_O_2_	−	+	−	−
	Benzoic acid	C_7_H_6_O_2_	−	−	+	−
	Biphenyl	C_12_H_10_	−	−	+	+
	Benzoic acid, phenyl ester	C_13_H_10_O_2_	−	−	+	+
	Benzaldehyde, 2,4,6-trimethyl- (mesitaldehyde)	C_10_H_12_O	−	+	−	−
Plasticizer/processing aid	2-Butenedioic acid (E)-, bis(2-ethylhexyl) ester	C_20_H_36_O_4_	+	−	−	−
	Hexadecanoic acid, methyl ester	C_17_H_34_O_2_	+	−	−	−
	Tributyl acetylcitrate	C_20_H_34_O_8_	−	−	+	−
	1-Butanol	C_4_H_10_O	−	−	+	+
Stabilizer/additive	Drometrizole	C_13_H_11_N_3_O	+	−	−	−
	1-Dodecene	C_12_H_24_	+	−	−	−
	1-Dodecanethiol	C_12_H_26_S	+	−	−	−
	Mequinol	C_7_H_8_O_2_	−	+	−	−
Terpene constituent	γ-Terpinene	C_10_H_16_	−	−	+	−
	o-Cymene	C_10_H_14_	−	−	+	+
Residual solvent	1-Methoxy-2-propyl acetate	C_6_H_12_O_3_	−	+	−	−
	Nonane	C_9_H_20_	−	+	−	−
Process-related impurity/by-product	Methyl isobutyrate	C_5_H_10_O_2_	−	−	+	+
Unassigned (no attributable source)	C_6_H_12_O species (library match: oxetane, 2,2,4-trimethyl-)	C_6_H_12_O	−	−	+	−
	N-/S-containing species (library match: acetic acid, 5-acetoxy-4-nitrotetrahydrothiopyran-3-yl ester)	C_9_H_13_NO_6_S	−	−	+	−
	C_4_H_6_O_3_ species (library match: propanoic acid, 2-oxo-, methyl ester)	C_4_H_6_O_3_	−	−	−	+

*+*,* compound detected by GC–MS in the corresponding solid specimen; −*,* not detected. Compounds are grouped by category (poly-/monomer*,* initiator/degradation product*,* plasticizer/processing aid*,* stabilizer/additive*,* terpene constituent*,* residual solvent*,* process-related impurity/by-product*,* and unassigned)*,* in accordance with the attribution given in Table **S1*,* and listed for each of the four materials (PreP*,* PP*,* AP*,* TP). A comparison of these findings with the manufacturer-declared ingredients is provided in Table **S1**. All compound names are tentative library annotations (NIST 2019) that were not confirmed by retention indices or reference standards. Compounds listed as unassigned could*,* in addition*,* not be attributed to a declared constituent*,* a known impurity*,* or a known degradation product of the respective material; the library name is given for traceability only and is not asserted as an identification.*

The substances detected in the solid specimens via GC–MS were compared with the manufacturer specifications (Table S1). This comparison is based on manufacturer details and the known chemistry of conventional PMMA-based polymerization, in which methyl methacrylate is polymerized using an initiator, while regulators influence the polymerization process. Plasticizers and further additives, including pigments and stabilizers, modify processing or material properties.

## Discussion

The present study examined the influence of the polymerization method and material formulation on residual monomer content and leachable substance profiles of four methacrylate-based denture base resins, two conventionally processed and two CAD/CAM-fabricated. CAD/CAM-based fabrication encompasses two distinct approaches: subtractive manufacturing, in which an industrially prepolymerized resin blank is milled into the desired prosthetic shape using a CNC unit, and additive manufacturing. Both approaches differ fundamentally from conventional polymerization not only in their processing conditions but also in their base monomer systems and degree of conversion, which has direct implications for the nature and quantity of leachable substances, a topic of increasing relevance as CAD/CAM technologies gain clinical adoption.

Before interpreting the findings, several methodological considerations warrant acknowledgment. The GC–MS-based quantification employed here was semi-quantitative, with results expressed as toluene-D8 equivalents; consequently, absolute concentration values should be treated with appropriate caution, and rank-order comparisons across materials are likely to be more informative than direct numerical contrasts. Furthermore, the single 48-hour static extraction conducted at 37 °C represents a considerable simplification of clinical reality, as it neither captures the cumulative effects of multi-year intraoral wear nor accounts for the thermo-mechanical loading cycles to which restorative materials are routinely subjected. These factors may lead to an underestimation of long-term release [37]. While KGM+ cell culture medium was selected as the extraction medium on account of its compatibility with downstream epithelial-cell assays, it does not fully recapitulate the biochemical complexity or dynamic flow characteristics of human saliva, which may influence both the extent and the profile of compound release [38, 39]. As MMA, the principal residual monomer of the conventional materials, was not released above the level of the negative control, cytotoxicity testing of the extracts was not performed in the present study; given the detection of other potentially bioactive compounds in the solid specimens and the limited coverage of high-molecular-weight dimethacrylates by the applied method, such testing remains a necessary next step.

One annotation warrants explicit comment. A low-abundance C_6_H_12_O species eluting shortly after MMA was detected exclusively in the autopolymerized specimens and was annotated by library match as 2,2,4-trimethyloxetane, a structure for which no plausible formation route from the declared constituents, including 1,4-butanediol dimethacrylate (BDDMA), could be identified. As the signal was absent from all other materials, a system-related contamination is unlikely, and it is regarded as material-derived; however, given its low abundance and the limited spectral specificity among the numerous C_6_H_12_O isomers, the library annotation is not considered reliable. This signal is therefore reported as unassigned (Table 1), as are the low-abundance C_4_H_6_O_3_ species in the thermally polymerized specimens and the nitrogen- and sulfur-containing species in the autopolymerized specimens, for which comparable considerations apply.

The compositional diversity of the tested materials necessitates that findings be interpreted within the context of each material’s monomer system. UDMA- and Bis-EMA-based systems are known to produce distinct leachable profiles, with network density and cross-linking architecture governing the extent and identity of eluted compounds [40, 41]. Because PP is formulated with Bis-EMA, UDMA, and TMPTMA rather than MMA/PMMA, it provides a compositional contrast that contributes to its distinct leachable profile. Accordingly, MMA was not expected and not detected in PP. It must be emphasized, however, that the PP system is based predominantly on high-molecular-weight dimethacrylates. Bis-EMA is of very low volatility and is therefore unlikely to elute under the applied GC conditions, and UDMA, which contains thermally labile urethane groups, may partially decompose at the injection temperature of 300 °C. Neither compound is therefore reliably captured by the present GC–MS screening, and their absence from the PP profile should not be interpreted as a general absence of leachable cross-linkers. In contrast, the trifunctional cross-linker TMPTMA (M ≈ 338 g/mol) is generally amenable to GC analysis. Its absence from the PP profile therefore more likely reflects a low residual content in the cured material than a methodological limitation, although a concentration below the detection limit of this semi-quantitative screening cannot be excluded. A targeted liquid-phase analysis (e.g., HPLC or LC–MS/MS) would be more appropriate for quantifying Bis-EMA and UDMA and represents the logical next step.

Surface roughness was comparable among all polished specimens (Ra ≈ 0.21–0.25 μm) but differed markedly on the unpolished, tissue-contacting surfaces (Ra 0.37–1.03 μm). As each extracted specimen comprised one polished and one unpolished surface, a contribution of surface roughness to compound release cannot be excluded; however, roughness was not evaluated as an independent factor, and no MMA release above the level of the negative control was detected in any group. Rougher surfaces, particularly those on the unpolished tissue-contacting side of dentures, are associated with increased monomer release, bacterial adhesion, and biofilm formation [32]. Clinically, this may contribute to microbial dysbiosis, particularly in immunocompromised or elderly patients [42], potentially exacerbating inflammation [42]. Beyond their potential to compromise biocompatibility, methacrylate-based monomers have also been investigated as carriers for antimicrobial activity through copolymerization with quaternary ammonium-based compounds, highlighting the dual role of monomer composition in both biological risk and functional material design [43–45].

GC–MS analysis of the solid specimens revealed that residual monomer content varied by polymerization method. Among the MMA/PMMA-based materials, thermal polymerization (TP) yielded the highest MMA content, followed by autopolymerized (AP) and prepolymerized (PreP) specimens.

GC–MS analysis of the corresponding extracts revealed no relevant MMA release into the extraction medium under the conditions applied, and extract data were not subjected to further quantitative interpretation. This finding is consistent with reports from studies using aqueous extraction media, in which monomer leaching from conventionally processed and CAD/CAM-fabricated materials was frequently below detection limits under short-term conditions [30, 46]. The relationship between residual monomer content in the solid phase and leachability into biological media therefore remains to be characterized under conditions that more closely replicate clinical exposure, including longer extraction periods and mechanical loading.

In the PP solid specimens, no methacrylate monomers were detected; however, other compounds were detected, including mesitaldehyde, mequinol, and 2,4,6-trimethylbenzoic acid. Mesitaldehyde and 2,4,6-trimethylbenzoic acid have been reported as degradation products of the photoinitiator diphenyl(2,4,6-trimethylbenzoyl)phosphine oxide (TPO), with both identities confirmed by authentic reference standards [47]. Because TPO is a manufacturer-declared component of PP, the corresponding library annotations are consistent with TPO-related degradation products; however, their identities and specific origin in the present specimens were not independently confirmed. Mequinol (4-methoxyphenol), a common stabilizer of methacrylate monomers [47], has been detected in extracts of a 3D-printed intraoral appliance material and reduced the viability of human gingival fibroblasts at higher concentrations [48]; it has further been shown to affect growth, metabolism, and inflammatory responsiveness of these cells in vitro [49]. Their mass fractions (mesitaldehyde 447 mg/kg, mequinol 209 mg/kg, 2,4,6-trimethylbenzoic acid 49 mg/kg; Figure S1) were obtained by the same semi-quantitative approach as the methacrylates but were not verified against authentic reference standards, so firm conclusions regarding their clinical significance cannot be drawn from the present data.

Comparison of manufacturer-provided compositional data with GC–MS findings revealed that GC–MS detected a broader range of substances than those declared by the manufacturers (Table S1). This discrepancy underscores the importance of independent analytical characterization as a complement to manufacturer disclosure.

Within the MMA/PMMA-based materials, the residual MMA content of the solid phase was lowest for the industrially prepolymerized, CAD/CAM-milled resin; for the 3D-printed resin, the absence of MMA follows from its MMA-free formulation and cannot be attributed to the fabrication route. This is in line with the observation that industrially prepolymerized milling blanks undergo controlled, high-conversion polymerization under optimized factory conditions, whereas chairside and laboratory polymerization techniques are more susceptible to variability in temperature, pressure, and duration [30, 50]. Low monomer values in the solid specimens are noteworthy, but the presence of other detected compounds with potential biological activity, such as drometrizole [51] or mequinol [52], indicates that a further, method-complementary toxicological evaluation of extractable substances is necessary before definitive conclusions regarding biocompatibility can be drawn.

Manufacturer-recommended post-polymerization procedures, such as water immersion, have been shown to reduce leachable content effectively [53]. Adherence to these recommendations in the present study may partly account for the low MMA concentrations observed in the biological extracts and underscores the importance of consistent application of such protocols in both clinical and laboratory settings. For 3D-printed materials in particular, post-processing variables including washing time, washing solution, and post-cure duration have been shown to exert a significant and independent effect on biocompatibility outcomes, with inadequate post-processing representing a major source of cytotoxicity [54]. However, these procedures are inconsistently applied across laboratory studies and are sometimes omitted, which may contribute to discrepancies in reported biocompatibility data.

## Conclusion

Fabrication method and material formulation jointly determine the residual monomer content and extractable substance profile of the tested denture base resins, rather than polymerization method acting as an isolated variable. Among the PMMA-based materials, residual monomer content in the cured material varied with fabrication route, being lowest for the CAD/CAM-milled and highest for the thermally polymerized specimens, yet MMA released into the extraction medium remained negligible under the tested short-term conditions for all groups. The 3D-printed resin showed a distinct set of detected constituents consistent with its formulation containing Bis-EMA, UDMA, and TMPTMA; because the present GC–MS screening may not reliably capture Bis-EMA and UDMA, their absence from the GC–MS results should not be interpreted as evidence that they are not leachable. Surface roughness was comparable among materials on the polished surfaces but differed on the unpolished, tissue-contacting surfaces.

Together, these findings indicate low MMA release under the tested short-term conditions while highlighting the need for longer-term, saliva-based, and complementary liquid-chromatographic investigations before their unreserved clinical adoption.

## Supplementary Information

Below is the link to the electronic supplementary material.

Supplementary Material 1

Supplementary figure 3 (PNG 755 KB)

High Resolution Image (TIFF 3.04 MB)

## Data Availability

All data supporting the findings of this study are available within the paper and its Supplementary Information.
